# Long-term Safety of Testosterone and Growth Hormone Supplementation: A Retrospective Study of Metabolic, Cardiovascular, and Oncologic Outcomes

**DOI:** 10.4021/jocmr428w

**Published:** 2010-08-18

**Authors:** Enrique Ginzburg, Nancy Klimas, Chad Parvus, Jeff Life, Robert Willix, Michale J. Barber, Alvin Lin, Florence Comite

**Affiliations:** a University of Miami Miller School of Medicine, Miami, Florida, USA; b Florida International University, Florida, USA; c Cenegenics Medical Institute, Las Vegas, Nevada, USA

## Abstract

**Background:**

Clinical research into the effects of hormonal supplementation has tended to focus on beneficial changes in anthropometric measures. There are fewer data on long-term safety with extended hormonal supplementation.

**Methods:**

As part of a retrospective database survey, clinical outcomes were tabulated among patients who received at least 1 year of testosterone and/or growth hormone (GH) supplementation. In patients who were treated for at least 2 years, changes in markers of glucose and lipid metabolism were analyzed with and without concomitant use of oral hypoglycemics and statins.

**Results:**

In 263 patients (mean age 56) treated for at least 2 years, the only statistically significant effect on markers of glucose metabolism was an increase in glycated hemoglobin (still within normal limits) in patients receiving GH alone or in combination with testosterone but without oral hypoglycemics; with or without hypoglycemics, insulin levels showed no significant change. The only significant effects on markers of lipid metabolism were decreases in total cholesterol and low-density lipoprotein (LDL) in patients receiving combined testosterone and GH without statins. Decreases in LDL were significant in both the statin and non-statin groups; decreases in triglycerides were significant only in the statin group. In 531 patients treated for at least 1 year (mean age 54), the overall incidence of adverse clinical outcomes (prostate disease, diabetes, cardiovascular disease, cancer) was 1.3%.

**Conclusions:**

In this retrospective survey, extended testosterone and/or GH supplementation did not adversely affect metabolic markers or clinical outcomes.

**Keywords:**

Safety; Testosterone; Growth hormone; Supplementation

## Introduction

To date, most investigations of the use of testosterone and growth hormone (GH) supplementation have focused on anthropometric measures of body composition (lean mass, fat mass, bone mineral density [BMD]) and sexual function in hypogonadal men with clinically significant testosterone deficiency. In these patients, supplementation has produced beneficial effects not only on libido and sexual function [1] but also on lean and fat mass [2]. Furthermore, in a small open trial in eugonadal men with idiopathic osteoporosis, testosterone supplementation was reported to produce notable increases in BMD [3]. A review of the literature on this topic confirms that normalization of testosterone can lead to a more favorable anthropometric profile [4].

Although favorable changes in anthropometric measures may be associated with decreases in the risk of osteoporotic fracture, coronary artery disease and type-2 diabetes mellitus, these clinical outcomes are more difficult to demonstrate, especially in the case of hormonal supplementation given to men whose testosterone levels are near normal rather than markedly deficient. In this setting, the clinical data are inconsistent. For example, in elderly men with low-to-normal testosterone, a non-controlled trial (N = 122) of testosterone with and without growth hormone (GH) supplementation reported increases in lean mass and decreases in fat mass accompanied by increases in muscle strength and aerobic endurance [5] whereas a placebo-controlled 6-month trial (N = 237) showed that supplementation produced the same pattern of favorable changes in lean and fat mass but no improvement in mobility or strength, or for that matter in BMD [6]. Testosterone supplementation in androgen-deficient women (often associated with hypopituitarism) is unusual, but the scant available data suggest that it may be beneficial in terms of sexual function, BMD, and lean mass, although adverse effects limit its clinical usefulness [7].

Similarly, while there is no major dispute with GH supplementation in individuals with documented GH deficiency, the benefit of supplementation in patients with near-normal GH levels remains controversial. In a small study of healthy elderly men participating in a program of resistance training, improvements in BMD were similar with and without adjunctive GH supplementation [8]. In contrast, adolescent males with idiopathic short stature and normal GH who received supplementation showed increased lean mass and decreased fat mass, along with favorable effects on lipids, but unfavorable increases in fasting insulin levels and hepatic gluconeogenesis [9].

While the debate continues over the benefits of hormonal supplementation in patients with near-normal endogenous hormone levels, there has been even less certainty about the long-term safety of supplementation, especially in terms of metabolic, cardiovascular, and cancer risk. The present report is a follow-up to a previously published database study showing that supplementation with testosterone, GH, or both had generally favorable effects on anthropometric measures in an outpatient population with low hormonal levels at baseline [10]; the focus here is on safety outcomes in that population.

## Methods

This retrospective database study examined the records of patients treated at the Cenegenics Medical Institute (Las Vegas, NV) during the period 1999-2006.

Table 1 summarizes the hormonal regimens used; in addition to the use of testosterone and GH, other hormonal treatments were provided as needed to achieve normalization of clinical and laboratory status, and all patients were advised to follow an exercise program and a low-glycemic diet.

Safety outcomes were assessed in 2 ways. Changes in metabolic markers were analyzed in patients who received hormonal treatment with testosterone (Tes), GH, or both (Tes+GH) for at least 2 years. These assessments included markers of glucose metabolism in patients who were and were not taking concomitant oral hypoglycemic agents for hyperglycemia or diabetes, and markers of lipid metabolism in patients who were and were not taking concomitant statins (HMG-CoA reductase inhibitors) for hyperlipidemia. In addition, treatment-emergent cases of cardiovascular disease, diabetes, cancer, and prostate disease were tabulated in the larger group of patients who had received hormonal treatment for at least 1 year.

Within-group mean changes from baseline to endpoint were assessed with a 2-sample t-test assuming equal variances. Between-group differences at a given time-point were assessed using the Mann-Whitney U test and the Kruskal-Wallis test. For correlations, Pearson's test was used for normally distributed data; otherwise, the Spearman rank test was used. All hypothesis tests were two-tailed, with statistical significance assessed at *P* < 0.05 with 95% confidence intervals. The statistical software used was SPSS 11.5 for Windows (SPSS Inc, Chicago, IL, USA).

**Table 1:** Hormonal Regimens

Therapy (route)	Goal	Measurement
Testosterone, men (intramuscular)*	Concentration representing 66th percentile (±12.5%) for 40-year-old men	Total 700 - 900 ng/dL, free 130 - 200 pg/mL
Testosterone, women (transdermal or sublingual)	Upper 33% of normal range for premenopausal women	Total 52-70 ng/dL
Human growth hormone (subcutaneous)	Upper 40% of normal range for age 39-54 years	Rise of ≥ 100% in insulin-like growth factor-1, to a maximum of 360 ng/mL

*Also includes use of human chorionic gonadotropin to increase endogenous testosterone production

## Results

### Patient populations

In all, the records of 531 patients (89% males, mean age 54 years) who received hormonal therapy for at least 1 year were reviewed. Of these, 24 patients received hormonal regimens other than the 3 that were the focus of this study. Of the remaining 507 patients, 282 were in the Tes group (receiving testosterone, human chorionic gonadotropin [hCG] to stimulate endogenous testosterone production, or both), 22 were in the GH group (receiving GH alone), and 203 were in the Tes+GH group (receiving GH in combination with testosterone and/or hCG). The records of 263 patients (mean age 56 years) who were treated for at least 2 years were assessed for changes in metabolic markers; however, because of the retrospective nature of this study, the numbers of patients in whom initial and final data on metabolic markers were available varied with each outcome measure. The records of all 531 patients who received hormonal treatment for at least 1 year were assessed for new-onset cardiovascular disease, diabetes and prostate disease. The mean duration of hormonal supplementation was approximately 18 months.

### Metabolic outcomes

Table 2 summarizes changes in markers of glucose metabolism over the course of 2 years of treatment in patients who were not receiving concomitant treatment with oral hypoglycemic agents. The only statistically significant change was an increase from the initial assessment to the final assessment in glycated hemoglobin (HbA_1c_) in the GH and Tes+GH groups; however, the final values (5.4% in both groups) were still well within normal limits.

Table 3 compares changes in insulin, as a representative marker of glucose metabolism during treatment with Tes+GH, in patients who did versus did not require concomitant treatment with oral hypoglycemics (note that the data shown for the patients not taking oral hypoglycemics match the corresponding data for insulin effects in the Tes+GH group as shown in Table 2). Although the change from initial to final insulin values appears substantial in the group receiving concomitant hypoglycemics, the number of patients involved was too small (n = 10) to establish statistical significance. However, the difference in insulin levels between groups was significant at the initial assessment and again at the final assessment, as would be expected between patients who do versus do not require therapy with oral hypoglycemic agents.

Table 4 shows the changes in markers of lipid metabolism in patients who were not receiving concomitant treatment with statins. Levels of total cholesterol and low-density lipoprotein cholesterol (LDL) fell from the initial assessment to the final assessment in all treatment groups, but the changes on both measures were statistically significant only in the Tes+GH group. Triglyceride levels also fell in all treatment groups; none of the changes was statistically significant, but the decrease approached significance in the Tes+GH group (*P* = 0.058), which started at a substantially lower mean value than the other groups (114 vs 134-136 mg/dL). High-density lipoprotein cholesterol (HDL) levels showed little change over the course of 2 years of treatment.

Table 5 compares the changes in HDL, LDL, and triglycerides during treatment with Tes+GH, in patients who did versus did not require concomitant treatment with statins. HDL showed little change in either group (with versus without concomitant statins), and there were no significant between-group differences at the initial assessment or at the final assessment. In contrast, LDL showed highly significant decreases from initial to final assessment in both groups, but with no significant between-group differences at either assessment. Triglyceride levels decreased in both groups, and the change from initial to final assessment, which only approached statistical significance in the non-statin group, was significant (*P* < 0.05) in the statin group. The greater magnitude of change in the statin group would be expected in patients receiving treatment for hyperlipidemia, and triglyceride levels were in fact higher in the statin group than in the non-statin group at both the initial and the final assessments. The between-group difference was significant only at the initial assessment, and the smaller difference at the final assessment reflects the results of statin therapy in lowering triglyceride levels.

**Table 2:** Markers of Glucose Metabolism in Patients Not Receiving Concomitant Oral Hypoglycemic Agents

Marker	Treatment	N	Initial Value	Final Value	*P* value*
Fasting blood glucose (mg/dL)	Tes	39	90	93	0.549
	GH	53	92	89	0.395
	Tes+GH	82	90	92	0.177
Insulin (μIU/mL)	Tes	39	6.5	5.4	0.372
	GH	51	5.7	7.3	0.137
	Tes+GH	75	5.7	5.8	0.893
HbA_1c_ (%)	Tes	47	5.4	5.5	0.964
	GH	57	5.1	5.4	< 0.001
	Tes+GH	90	5.1	5.4	< 0.001

Tes: supplementation with testosterone (including use of human chorionic gonadotropin [hCG]); GH: supplementation with human growth hormone; Tes+GH: supplementation with testosterone and/or hCG, in combination with human growth hormone; HbA_1c_: glycated hemoglobin. *Change from initial to final value within treatment group.

**Table 3:** Marker of Glucose Metabolism In Patients Treated With Tes+GH With Versus Without Concomitant Oral Hypoglycemic Agents

Marker	Treatment: Tes+GH	N	Initial Value	Final Value	*P* value*
Insulin (μIU/mL)	With hypoglycemics	10	9.2	12.3	0.326
	Without hypoglycemics	75	5.7	5.8	0.893
*P* value**			< 0.005	< 0.001	

Tes+GH: supplementation with testosterone and/or human chorionic gonadotropin, in combination with human growth hormone. *Change from initial to final value within treatment group. **Difference between treatment groups at initial assessment and at final assessment.

**Table 4:** Markers of Lipid Metabolism in Patients Not Receiving Concomitant Statins

Marker	Treatment	N	Initial Value	Final Value	*P* value*
Cholesterol (mg/dL)	Tes	35	224	177	0.155
	GH	7	204	198	0.759
	Tes+GH	106	208	183	< 0.001
HDL (mg/dL)	Tes	39	48	48	0.947
	GH	7	47	56	0.286
	Tes+GH	113	55	54	0.729
LDL (mg/dL)	Tes	39	115	105	0.116
	GH	7	125	115	0.597
	Tes+GH	114	129	108	< 0.001
Triglycerides (mg/dL)	Tes	39	134	121	0.483
	GH	7	136	110	0.636
	Tes+GH	114	114	97	0.058

Tes: supplementation with testosterone (including use of human chorionic gonadotropin [hCG]); GH: supplementation with human growth hormone; Tes+GH: supplementation with testosterone and/or hCG, in combination with human growth hormone; HDL: high-density lipoprotein cholesterol; LDL: low-density lipoprotein cholesterol. *Change from initial to final value within treatment group.

**Table 5:** Markers of Lipid Metabolism in Patients Treated With Tes+GH With Versus Without Concomitant Statins

Marker	Treatment: Tes+GH	N	Initial Value	Final Value	*P* value*
HDL (mg/dL)	With statins	35	52	53	0.844
	Without statins	113	55	54	0.729
*P* value**			0.373	0.719	

LDL (mg/dL)	With statins	35	137	97	< 0.001
	Without statins	114	129	108	< 0.001
*P* value**			0.267	0.122	

Triglycerides (mg/dL)	With statins	35	154	110	< 0.05
	Without statins	114	114	97	0.058
*P* value**			< 0.05	0.255	

Tes+GH: supplementation with testosterone and/or human chorionic gonadotropin, in combination with human growth hormone; HDL: high-density lipoprotein cholesterol; LDL: low-density lipoprotein cholesterol. *Change from initial to final value within treatment group. **Difference between treatment groups at initial assessment and at final assessment.

### Clinical outcomes

Among 531 patients who received at least 1 year of hormonal treatment (including 24 who were on regimens other than Tes, GH, or Tes+GH), 7 (1.3%) experienced new disease events during the study period. These treatment-emergent outcomes comprise 1 cardiac event, 1 case of hypertension, 2 cases diabetes, and 3 cases of nonmalignant prostatic disease. There were no new cases of malignancy of any kind reported during the study period.

A total of 110 patients (20.7%) had pre-existing disease (including, for example, diabetes, which would account for the use of oral hypoglycemic drugs during hormonal supplementation; or hyperlipidemia, which would account for the use of statins). Within this subgroup, 63 (57.3%) experienced no notable change in their pre-existing conditions, 39 (35.5%) reported improvement, and 8 (7.3%) reported worsening.

## Discussion

This retrospective database study showed that hormonal supplementation with testosterone or GH or both, in conjunction with beneficial lifestyle changes in diet and exercise, was generally safe in terms of risk of adverse changes in metabolic markers and adverse clinical outcomes.

Our findings must be placed in perspective with the extant literature on safety concerns with hormonal supplementation. Looking first at testosterone, an obvious concern would be supplementation in men at increased risk for occurrence or recurrence of prostate cancer. The fact that castration has sometimes been employed to retard the advance of prostate cancer does not necessarily mean that testosterone supplementation in hypogonadal men increases their risk. Reviews on this subject dispute the assumption that risk is lower with depressed levels of testosterone [11, 12], and a recent comprehensive review finds no evidence that supplementation to normalize testosterone levels increases the risk of prostate cancer [13]. On the other hand, a survey of urology patients found a number of cases of new-onset prostate cancer that appeared within a few months to a few years of initiation of testosterone supplementation and were considered treatment-related [14]. Clearly, testosterone supplementation would not be appropriate in a patient in whom prostatic cancer is suspected or documented [15, 16].

With respect to cancer risk in women who receive testosterone supplementation (e.g., for low sexual desire), a review of the literature found inconsistent clinical data from flawed trials, and concluded that the available evidence does not clearly demonstrate any increased risk of breast cancer from adding testosterone to postmenopausal hormonal replacement therapy [17].

Diabetes risk may actually be reduced through testosterone supplementation. In men with diabetes, who tend to show lower-than-normal levels of circulating androgen, supplementation improves glucose homeostasis, reduces visceral fat, and corrects insulin resistance [18]. Other comprehensive reviews conclude that testosterone supplementation can be beneficial in reducing the risks of diabetes and metabolic syndrome in hypogonadal men [19, 20].

With respect to cardiovascular risk, a small study showed no adverse increases in atherogenic lipids in 7 hypogonadal men who completed 18 months of testosterone supplementation [21]. Similarly, no increased cardiovascular risk or harmful changes in atherogenic lipids were reported in the previously mentioned study of 23 eugonadal men who showed increased BMD after 6 months of testosterone treatment for idiopathic osteoporosis [3]. Far from increasing cardiovascular risk, testosterone supplementation may actually reduce risk in men (through beneficial effects on fibrinolysis, vascular endothelium, glucose metabolism, and coronary circulation), and potentially might also reduce the elevated cardiovascular risk seen in women following hysterectomy [22].

Turning to the smaller body of literature on GH, there are few reports relating to cancer risk. In a case report, colon cancer developed in a patient at increased risk due to Crohns colitis, following use of GH as anti-aging therapy [23]. In contrast, there was no evidence of tumorogenicity in a preclinical report of GH administration in a mouse model [24]. In addition, a study in healthy volunteers found that GH supplementation increased the antitumor activity of natural killer cells [25].

In another preclinical model involving mice fed a high-fat diet, low-dose GH supplementation seemed to inhibit the expression of cellular changes associated with insulin resistance [26], suggesting a possible protective effect.

A small study in 7 fit men with normal hormone levels showed that GH supplementation led to dose-related decreases in total cholesterol [27]. A more definitive study in obese men and women found that GH produced a modest reduction in body fat had no significant effect on markers of adiposity (leptin, adiponectin, and C-reactive protein); nevertheless, no worsening in metabolic markers was reported [28]. In a preclinical model of congestive heart failure, short-term use of GH improved left ventricular function through favorable effects on myocardial remodeling and contractility [29].

Not surprisingly, the overall conclusion from the literature is that whereas the medical benefits of hormonal supplementation can be clearly documented in hypogonadal patients, the proper role of supplementation is, at this time, harder to define in patients who are not clearly hypogonadal [30]. Such discrepancies as may exist between our study and previously published reports do not seem to involve directly contradictory findings.

Next, it is necessary to place the current findings in perspective with risks in the general population. The annual incidence of coronary heart disease is estimated at 0.3% among males in the age range 35-44 years; the incidence among women is somewhat lower and the overall incidence of cardiovascular disease of any kind would be higher [31]. Our study population might be considered at greater risk than the reference population on the basis of older age, and at slightly lower risk because it included women (11% of the population). Among 531 patients who received hormonal supplementation for at least 1 year and for a mean period of approximately 18 months, only 1 treatment-emergent case of cardiac disease was reported (incidence 0.2%). Even if the single case of treatment-emergent hypertension is also included in this category, the incidence of new-onset cardiovascular disease in our population, 2 cases among 531 patients (0.4%), is no higher than the incidence in the general population.

Similarly, the incidence of treatment-emergent diabetes in our population may be compared with the annual incidence of diabetes in the general population, which is estimated at 0.7% in people over age 20 years [32]. The age range of our study population lies within that reference range, and the 2 cases of new-onset diabetes we report represent an incidence of 0.4%, which, again, is certainly no higher than the incidence in the general population.

Finally, with respect to cancer risk, there were no cases of new-onset malignancy in our study. By contrast, within the general population, the annual incidence is approximately 0.4% (higher in men, lower in women) for all forms of cancer combined, excluding common basal and squamous cell carcinomas of the skin [33].

Although the incidence of these adverse outcomes might be expected to be higher in this more closely monitored population than in the general population, the reported incidence rates of cardiovascular disease, diabetes, and cancer over the course of at least 1 year of hormonal supplementation were, in fact, no higher and possibly lower than the 1-year incidence rates for these conditions in the general population. Indeed, the overall incidence of adverse clinical outcomes (7 among 531 patients, or 1.3%) is too small for any meaningful assessment of risk by treatment group.

However, comparisons of risk in our study population versus the general population must be interpreted with caution. For example, the fact that the incidence of cancer was zero in our population in no way supports a claim of decreased risk with hormonal supplementation. What these comparisons demonstrate is that long-term hormonal supplementation was not associated with any discernible increased risk.

The main limitations of this study are its design as a retrospective database survey and the lack of stratification of outcome data by clinical and demographic variables. The current findings do not distinguish between results in younger versus older patients, in male versus female patients, or in patients with different levels of endogenous hormones and different anthropometric measures of body composition at baseline. Similarly, among patients taking concomitant oral hypoglycemic or statin therapy, there is no comparison of changes in metabolic markers in those taking lower versus higher doses of concomitant medications for milder versus more severe pre-existing abnormalities in glucose or lipid metabolism. Furthermore, we cannot determine to what degree the healthful diet-and-exercise regimen may have modulated changes in metabolic markers and the risk of adverse clinical outcomes, or to what degree the reported treatment-emergent adverse clinical outcomes were treatment-related, that is, which events were possibly or probably attributable to hormonal supplementation. On the other hand, such analyses might not have yielded meaningful distinctions between subgroups given the low overall rate of adverse outcomes. Another limitation is that the number of patients treated with GH alone (n = 22) was much smaller than the numbers treated with testosterone (n = 282) or combined testosterone and GH (n = 203); therefore, the outcomes reported for GH cannot be considered as robust as the outcomes reported for the other regimens. Notwithstanding these limitations, our results, showing no evidence of increased clinical risk after 1 to 2 years of hormonal supplementation, may be relevant in light of the relative paucity of long-term data on safety outcomes after extended treatment with testosterone and GH.

### Conclusions

In this retrospective database survey, long-term hormonal supplementation with testosterone and/or human growth hormone was not associated with adverse effects on markers of glucose or lipid metabolism, and did not incur increased risk of adverse clinical outcomes relating to new onset of diabetes, cardiovascular disease, or cancer.

## References

[1] Perleth M (2007). Testosterone--a "fountain of youth" for ageing men? Brief assessment of the efficacy and safety of androgen substitution in healthy men. Z Arztl Fortbild Qualitatssich.

[2] Wittert GA, Chapman IM, Haren MT, Mackintosh S, Coates P, Morley JE (2003). Oral testosterone supplementation increases muscle and decreases fat mass in healthy elderly males with low-normal gonadal status. J Gerontol A Biol Sci Med Sci.

[3] Anderson FH, Francis RM, Faulkner K (1996). Androgen supplementation in eugonadal men with osteoporosis-effects of 6 months of treatment on bone mineral density and cardiovascular risk factors. Bone.

[4] Kohn FM (2006). Testosterone and body functions. Aging Male.

[5] Sattler FR, Castaneda-Sceppa C, Binder EF, Schroeder ET, Wang Y, Bhasin S, Kawakubo M (2009). Testosterone and growth hormone improve body composition and muscle performance in older men. J Clin Endocrinol Metab.

[6] Emmelot-Vonk MH, Verhaar HJ, Nakhai Pour HR, Aleman A, Lock TM, Bosch JL, Grobbee DE (2008). Effect of testosterone supplementation on functional mobility, cognition, and other parameters in older men: a randomized controlled trial. JAMA.

[7] Zang H, Davis SR (2008). Androgen replacement therapy in androgen-deficient women with hypopituitarism. Drugs.

[8] Yarasheski KE, Campbell JA, Kohrt WM (1997). Effect of resistance exercise and growth hormone on bone density in older men. Clin Endocrinol (Oxf).

[9] Hannon TS, Danadian K, Suprasongsin C, Arslanian SA (2007). Growth hormone treatment in adolescent males with idiopathic short stature: changes in body composition, protein, fat, and glucose metabolism. J Clin Endocrinol Metab.

[10] Ginzburg E, Lin A, Sigler M, Olsen D, Klimas N, Mintz A (2008). Testosterone and growth hormone normalization: a retrospective study of health outcomes. J Multidisc Healthcare.

[11] Raynaud JP (2006). Prostate cancer risk in testosterone-treated men. J Steroid Biochem Mol Biol.

[12] Morgentaler A (2006). Testosterone replacement therapy and prostate risks: where's the beef?. Can J Urol.

[13] Raynaud JP (2009). Testosterone deficiency syndrome: treatment and cancer risk. J Steroid Biochem Mol Biol.

[14] Gaylis FD, Lin DW, Ignatoff JM, Amling CL, Tutrone RF, Cosgrove DJ (2005). Prostate cancer in men using testosterone supplementation. J Urol.

[15] Holyoak JD, Crawford ED, Meacham RB (2008). Testosterone and the prostate: implications for the treatment of hypogonadal men. Curr Urol Rep.

[16] Brawer MK (2003). Androgen supplementation and prostate cancer risk: strategies for pretherapy assessment and monitoring. Rev Urol.

[17] Bitzer J, Kenemans P, Mueck AO (2008). Breast cancer risk in postmenopausal women using testosterone in combination with hormone replacement therapy. Maturitas.

[18] Fukui M, Kitagawa Y, Ose H, Hasegawa G, Yoshikawa T, Nakamura N (2007). Role of endogenous androgen against insulin resistance and athero- sclerosis in men with type 2 diabetes. Curr Diabetes Rev.

[19] Kalyani RR, Dobs AS (2007). Androgen deficiency, diabetes, and the metabolic syndrome in men. Curr Opin Endocrinol Diabetes Obes.

[20] Lunenfeld B (2007). Testosterone deficiency and the metabolic syndrome. Aging Male.

[21] Berg G, Schreier L, Geloso G, Otero P, Nagelberg A, Levalle O (2002). Impact on lipoprotein profile after long-term testosterone replacement in hypogonadal men. Horm Metab Res.

[22] Rako S (1998). Testosterone deficiency: a key factor in the increased cardiovascular risk to women following hysterectomy or with natural aging?. J Womens Health.

[23] Melmed GY, Devlin SM, Vlotides G, Dhall D, Ross S, Yu R, Melmed S (2008). Anti-aging therapy with human growth hormone associated with metastatic colon cancer in a patient with Crohn's colitis. Clin Gastroenterol Hepatol.

[24] Schubert R, Schmitz N, Pietzner J, Tandi C, Theisen A, Dresel R, Christmann M (2009). Growth hormone supplementation increased latency to tumourigenesis in Atm-deficient mice. Growth Factors.

[25] Crist DM, Kraner JC (1990). Supplemental growth hormone increases the tumor cytotoxic activity of natural killer cells in healthy adults with normal growth hormone secretion. Metabolism.

[26] Kubota Y, Unoki H, Bujo H, Rikihisa N, Udagawa A, Yoshimoto S, Ichinose M (2008). Low-dose GH supplementation reduces the TLR2 and TNF-alpha expressions in visceral fat. Biochem Biophys Res Commun.

[27] Crist DM, Peake GT, Loftfield RB, Kraner JC, Egan PA (1991). Supplemental growth hormone alters body composition, muscle protein metabolism and serum lipids in fit adults: characterization of dose-dependent and response-recovery effects. Mech Ageing Dev.

[28] Albert SG, Haas MJ, Mooradian AD (2007). The effects of recombinant human growth hormone (rhGH) supplementation on adipokines and C-reactive protein in obese subjects. Growth Horm IGF Res.

[29] Houck WV, Pan LC, Kribbs SB, Clair MJ, McDaniel GM, Krombach RS, Merritt WM (1999). Effects of growth hormone supplementation on left ventricular morphology and myocyte function with the development of congestive heart failure. Circulation.

[30] Yeap BB (2009). Testosterone and ill-health in aging men. Nat Clin Pract Endocrinol Metab.

[31] (2009). American Heart Association. Heart Disease and Stroke Statistics.

[32] (2007). National Diabetes Information Clearinghouse, National Institute of Diabetes and Digestive and Kidney Disease, National Institutes of Health.

[33] (2009). American Cancer Society. Cancer Statistics.

